# Unprepared and Misinformed Parents of Children with Sickle Cell Disease: Time to Rethink Awareness Campaigns

**DOI:** 10.7759/cureus.3806

**Published:** 2018-12-31

**Authors:** Seyi Aderotoye-Oni, Ijeoma N Diaku-Akinwumi, Adeyinka Adeniran, Bode Falase

**Affiliations:** 1 Internal Medicine, University of Pittsburgh Medical Center Pinnacle, Harrisburg, USA; 2 Paediatrics, Lagos State University Teaching Hospital, Lagos, NGA; 3 Epidemiology and Public Health, Lagos State University Teaching Hospital, Lagos, NGA; 4 Cardiothoracic Surgery, Lagos State University Teaching Hospital, Lagos, NGA

**Keywords:** sickle cell disease, anemia, public awareness, public health analysis, caregiver burden

## Abstract

Worldwide, sickle cell disease (SCD) poses a significant public health concern. It causes recurrent morbidity, and premature death is a distinct possibility, especially in Nigeria, which bears half the world’s burden of SCD patients. Nigeria has yet to establish a newborn screening program; consequently, most affected children are diagnosed between one and three years of age when a health problem arises. Parents are unprepared to identify SCD and seek comprehensive management early enough for the best outcome. Awareness of carrier status and knowledge of SCD would ensure informed reproductive choices.

Questionnaires were employed to conduct a prospective, cross-sectional study of the occurrence of missed carrier status of one biological parent of children enrolled at the pediatric sickle cell disease clinic of a teaching hospital. The institution’s ethics committee approved the study.

Of 133 respondents, 53% of the parents were unaware of being sickle cell carriers and did not expect to have children with SCD. Among families in which one or both parents proactively sought to know their genotype, 35% of all parents received incorrect genotyping results regarding the SCD risk of their offspring. The majority of incorrectly assigned hemoglobin AA results occurred in private laboratories. More than 60% of the respondents reported gaining awareness regarding SCD through antenatal care (51%) and schools (17%), as opposed to public awareness campaigns (8%).

Many parents in our study population were unprepared for their children’s SCD diagnosis. Awareness campaigns need to be revisited as a major potential modality to increase outreach about SCD. Standardization and improved quality control of laboratory testing involving screening of the sickle cell trait could reduce the frequency of wrong genotype assignment.

## Introduction

Sickle cell disease (SCD) is a global health problem affecting over 100 million people [1-2] and is most common in people of African descent. Every year, 300,000 babies of all races are born with sickle cell disease globally; half of those cases are in Nigeria [3]. Despite SCD being a predominant public health concern in Nigeria, the literature remains scarce and not uniformly available with regards to the exact mortality rates in Nigeria. Estimates suggest that 50%-80% of these patients will die before adulthood [4]. It has been estimated that about 2.3% of the Nigerian population suffer from the disorder and over 40 million Nigerians are carriers of the sickle cell gene, making it the most common inherited genetic disorder affecting Nigerians and, thus, a significant community health concern [5]. Every single day, Nigeria loses about 2,300 under five-year-olds and the under-five mortality rate stands at 104 per 1000 live births in 2016 [6].

SCD is an autosomal recessive disorder and can be clinically expressed either by receiving two copies of hemoglobin (Hb) S (sickle cell anemia) or one copy of HbS plus another β-globin variant (such as HbC, β-thalassemia, HbD, or HbE) [7]. Unlike the normal bi-concave disc shape of a red blood cell (RBC), the mutation of the β-globin gene results in the abnormal development of RBCs, causing them to become stiff, sticky, and sickle-shaped when deoxygenation prevails. Consequently, this affects the oxygen-carrying capacity of the cells and their ability to pass through vessels easily [8].

The presence of the HbS gene can be detected either prenatally or postnatally [3,9]. Prenatally, diagnosis can be made via chorionic villus sampling and amniocentesis; postnatally, the sickling test (determines carrier status), Hb electrophoresis, high-performance liquid chromatography (HPLC), isoelectric focusing (IEF), and DNA analysis can be used to diagnose SCD.

SCD interferes with many aspects of the patients' and their family members’ lives, including psychosocial development, education, marital condition, employment, and overall quality of life [10]. Genetic screening and counseling help ensure that parents make informed decisions regarding family planning and reproduction and have the opportunity to provide early preventive interventions to children with SCD.

The most common technique used to diagnose SCD and to identify sickle cell trait carriers is Hb electrophoresis [11]. In general, adults may have the following types of Hb: HbA 95% to 98%, HbA_2_ 2% to 3%, HbF 0.8% to 2%, HbS 0%, and HbC 0%. For infants and children, the Hb consists of the following types: HbF (newborn) 50% to 80%, HbF (six months) 8%, and HbF (over six months) 1% to 2%. Abnormal laboratory results signify the presence of possibly significant levels of abnormal HbS, which may indicate SCD and other hemoglobinopathies [12-13].

The average age at which the confirmation of the Hb genotype in children with SCD in Lagos, Nigeria, occurs is around 27.33 months. There are several essential implications associated with knowing one’s sickle cell trait status, ranging from ethical considerations (in vitro and in vivo genetic testing) to those concerning societal prejudices [14]. Due to a lack of prenatal diagnosis, three-quarters of the children with SCD are at risk of premature death due to delayed medical intervention [15-16].

The aim of our study was to determine the prevalence and correlation of missed sickle cell disease carrier identification among parents of children with SCD.

## Materials and methods

Study design

A prospective, one-center, cross-sectional study was performed at a pediatric sickle cell clinic. The clinic was in a tertiary health institution and is one of the main referral facilities providing both general and specialist pediatric care for inhabitants of one of the largest cities in West Africa. Our study was conducted over three months from January 2014 to March 2014. A semi-structured questionnaire, developed by the study team, was administered to 133 parents of children registered at the pediatric sickle cell clinic who were enrolled in the study.

Data regarding parents' age, level of education, family size, whether the parent’s genotype was known before the birth of their child with SCD, and the effect of having a child with SCD on various aspects of daily life, including finances, education, and family interaction, was collected. The biological parent(s) of the child(ren) presenting at the sickle cell clinic and those who have or had a child with SCD who is or was tested and registered at the sickle cell clinic were included in the study. The parents of the child(ren) with SCD presenting with life-threatening medical emergencies were excluded. During the appointments at the sickle cell clinic, the parents were explained the study and consent was taken.

Sample size calculation

N=Z^2^ p(1-p)/d^2^, where “N” is the minimum sample size, “Z” is the critical value in the two-tailed test, “p” is the expected proportion of wrong carrier statuses in the population, and “d” is the permissible standard error. Here, Z=1.96, p=8% and d=0.05. Therefore, N=1.962 x 0.08(0.092)/0.0025=113.096. If 10% is added for attrition, sample size becomes 124 patients. One hundred and thirty parents of children with SCD were studied. The calculations were based on a brief pilot study conducted by the authors at the same site.

Statistical methods

Continuous variables were reported as mean (standard deviation) and range and categorical variables as number (percent). The Chi-square test was used to analyze between-group differences for the categorical variables. A p-value of <0.05 was considered statistically significant. All the analyses were done using SAS 9.4 (SAS Institute, Cary, NC, US).

## Results

Age, sex, religion, and marital status of parents of children with SCD

A total of 133 parents of children with SCD were assessed during the study period. They consisted of 125 mothers (96%) and eight fathers (4%). The ages of the parents ranged from 23 to 55 years with a mean of 37.3 years. Ninety (67.7%) of the parents were Christian, and 43 (32.3%) were Muslim. None of the parents reported traditional practices as a religion. A majority of the parents interviewed were married (93.23%), three were widowed (2.3%), and two were single (1.5%).

Level of education and monthly income of the parents

A total of 52 parents (39.1%) had completed secondary education, 41 parents (30.8%) received tertiary level education, 18 parents (13.5%) had completed primary education, and 19 parents (14.3%) had completed a level of education considered as other. The majority of the parents (47, 35.3%) reported a monthly income of 10-30,000 naira, 32 parents (24.1%) reported 30-60,000 naira, 24 parents (18%) reported 60-100,000 naira, 16 parents (12%) reported more than 100,000 naira, and 14 parents (10.5%) reported less than 10,000 naira (Tables 1-4). Conversion to US dollars has not been included in the tables due to the economic variability and constant fluctuations in market exchange rates. The financial impact would be inadequately gauged by conversion against US dollars.

**Table 1:** Variation with monthly income (respondents)

Respondents Initial Genotype								
Monthly Income	Correct Freq. (%)		Misidentified Public Freq. (%)	Misidentified Private Freq. (%)		Unknown Freq. (%)		Total
<10k naira	3 (4.76%)		0 (0%)	1 (6.67%)		10 (19.23%)		14 (10.53%)
10-30k naira	17 (26.98%)		0 (0%)	5 (33.33%)		25 (48.08%)		47 (35.34%)
30-60k naira	16 (25.40%)		2 (66.67%)	3 (20%)		11 (21.15%)		32 (24.06%)
60-100k naira	15 (23.81%)		1 (33.33%)	2 (13.33%)		6 (11.54%)		24 (18.05%)
>100k naira	12 (19.05%)		0 (0%)	4 (26.67%)		0 (0%)		16 (12.03%)
Total	63 (47.37%)		3 (2.26%)	15 (11.28%)		52 (39.10%)		133 (100%)
Chi-Square Tests								
		Value			df		Asymp Sig. (2-sided)	
Pearson Chi-Square		28.384 (a)			12		.005	
Likelihood Ratio		34.246			12		.001	
No of Valid Cases		133						

**Table 2:** Variation with monthly income (spouse)

Spouse Initial Genotype					
Monthly Income	Correct Freq. (%)	Misidentified Public Freq. (%)	Misidentified Private Freq. (%)	Unknown Freq. (%)	Total
<10k naira	1 (3.22%)	0 (0%)	2 (6.67%)	11 (15.94%)	14 (10.53%)
10-30k naira	8 (25.51%)	1 (33.33%)	6 (20%)	32 (46.38%)	47 (35.34%)
30-60k naira	9 (29.03%)	0 (0%)	8 (26.67%)	15 (21.74%)	32 (24.06%)
60k-100k naira	7 (22.58%)	0 (0%)	8 (26.67%)	9 (13.04%)	24 (18.04%)
>100k naira	6 (19.35%)	2 (66.67%)	6 (20%)	2 (2.90%)	16 (12.03%)
Total	31 (23.31%)	3 (2.26%)	30 (22.56%)	69 (51.88%)	133 (100%)
Chi-Square Tests					
	Value	df	Asymp. Sig. (2- sided)		
Pearson Chi-Square	28.969	12	.004		
Likelihood Ratio	28.851	12	.004		
No of Valid Cases	133				

**Table 3:** Variation with education (respondents) (a) 11 cells (55.0%) have expected count less than 5. The minimum expected count is .07. Primary: First seven to nine years of formal education Secondary: Last four years of formal education Tertiary: College and graduate schools Other: Certificate programs after secondary school

Respondents Initial Genotype								
Educational Level	Correct Freq. (%)		Misidentified Public Freq. (%)	Misidentified Private Freq. (%)		Unknown Freq. (%)		Total
None	0 (0%)		0 (0%)	0 (0%)		3 (5.8%)		3 (2.3%)
Primary	5 (7.9%)		1 (33.3%)	1 (6.7%)		11 (21.2%)		18 (13.5%)
Secondary	18 (28.6%)		0 (0%)	4 (26.7%)		30 (57.7%)		52 (39.1%)
Tertiary	28 (44.4%)		2 (66.7%)	6 (40%)		5 (9.6%)		41 (30.8%)
Other	12 (19%)		0 (0%)	4 (26.7%)		3 (5.8%)		19 (14.3%)
Total	63 (47.4%)		3 (2.3%)	15 (11.3%)		52 (39.1%)		133 (100%)
Chi-Square Tests								
		Value			df		Asymp Sig. (2-sided)	
Pearson Chi-Square		36.621 (a)			12		.000	
Likelihood Ratio		41.006			12		.000	
No of Valid Cases		133						

**Table 4:** Variation with education (spouse) (a) 12 cells (60.0%) have expected count less than 5. The minimum expected count is .07.

Spouse Initial Genotype								
Educational Level	Correct Freq. (%)		Misidentified Public Freq. (%)	Misidentified Private Freq. (%)		Unknown Freq. (%)		Total
None	0 (0%)		0 (0%)	0 (0%)		3 (4.3%)		3 (0.3%)
Primary	3 (9.7%)		0 (0%)	1 (3.3%)		14 (20.03%)		18 (13.5%)
Secondary	9 (29%)		2 (66.7%)	7 (23.3%)		34 (49.3%)		52 (39%)
Tertiary	15 (48.4%)		1 (33.33%)	16 (53.3%)		9 (13%)		41 (30.8%)
Other	4 (12.9%)		0 (0%)	6 (20%)		9 (13%)		19 (14.3%)
Total	31 (23.3%)		3 (2.3%)	30 (22.3%)		69 (51.9%)		133 (100%)
Chi-Square Tests								
		Value			df		Asymp Sig. (2-sided)	
Pearson Chi-Square		29.630 (a)			12		.003	
Likelihood Ratio		33.070			12		.001	
No of Valid Cases		133						

When and where parent first learned about SCD

The parents who knew about SCD before the birth of their child with SCD (67, 50.4%) was almost evenly split with those parents who learned about SCD after the birth of a child with SCD (66, 49.6%). A majority of the parents (79, 50.6%) learned about SCD via the hospital, 27 parents (17.3%) learned about SCD via school, 26 parents (16.7%) learned via friends, neighbors, etc., 11 parents (7.1%) learned from family, 11 parents (7.1%) learned from media, and two parents (1.2%) learned about SCD from church SCD campaigns.

Reason parents got their initial genotype tested

Twenty-three parents (33.8%) initially did their genotyping due to school requirements, 14 (20.6%) due to an awareness of the disease, 13 (19.1%) due to premarital counseling, 10 (14.7%) as part of their antenatal care (ANC), four (5.9%) due to a family history of SCD, and four (5.9%) because of church/mosque SCD campaigns.

Currently reported genotype of the respondent parent

All 133 respondents reported a current genotype of sickle cell trait (AS) (100%), with 70 (52.6%) tested in a private lab while 63 (47.4%) were tested in a public lab.

Currently reported genotype of the second parent

The respondents also reported on their spouse’s/second-parent genotype status. The majority (130, 97.7%) were reported to be AS, one parent HbC trait (AC) (0.75%), one parent sickle cell HbC disease (SC) (0.75%), and one parent sickle cell anemia (SS) (0.75%). Sixty-nine of those (51.9%) were reported to have done their genotype in a private lab while 64 parents (48.1%) reported having completed their genotype in a public lab.

Genotype initially assigned to respondent

A total of 63 respondents (47.4%) reported to have known their correct genotype, 18 respondents (13.5%) reported to have been misidentified as normal Hb (AA); of those, three respondents (2.3%) had their genotype done in a public lab, and 15 respondents (11.3%) had their genotype done in a private lab. A total of 69 respondents (51.9%) genotypes were unknown before the marriage or conception of their child with SCD.

Genotype initially assigned to the second parent

A total of 31 spouses/second parents (23.3%) were reported to have known their correct genotype initially; 33 parents (24.8%) were misidentified as AA; of those, three parents (2.3%) carried out the genotype in a public lab while 30 parents (22.6%) conducted their exam in a private lab. Over half of the spouses/second parents (69 parents, 51.9%) were reported not to have known their genotype before marriage or conception of their child with SCD (refer to Table 5 for the combined figures of respondents and second parent). The total n=266 is a reflection of the total amount of parents. Though only one parent completed the questionnaire, information like their genotype for their spouse could be counted, thus, a total of 266 rather than 133 in Table 5.

**Table 5:** Initially assigned genotype (respondent (N=133) + spouse (N=133)) Total percent of misidentified genotypes is 19.2%.

	Responses			
	N		Percent	
Correctly identified		94	35.3	
Misidentified as AA (Pub)		6	2.3	19.2
Misidentified as AA (Priv)		45	16.9	
Unknown		121	45.5	
Total		266	100	

Consideration of genotype before marriage or conception

The majority of the respondents (95, 71.4%) reported that they did not consider their genotype nor their partners' before entering into marriage or conceiving their child with SCD while 38 did (Table 6).

**Table 6:** Genotypes taken into consideration before marriage/conception (a) 0 cells (0%) have expected count less than 5. The minimum expected count is 8.86.

			Initial Genotype (Respondents + Spouse)			
Genotypes taken into consideration before marriage/conception			Correct Freq. (%)	Misidentified Freq. (%)	Unknown Freq. (%)	Total
Yes			10 (32.3%)	24 (72.7%)	4 (5.8%)	38 (28.6%)
No			21 (67.7%)	9 (27.3%)	65 (94.2%)	95 (71.4%)
Total			31 (23.3%)	33 (24.8%)	69 (51.9%)	133 (100.0%)
Chi-Square Tests						
	Value	df		Asymp. Sig. (2-sided)		
Pearson Chi-Square	49.270 (a)	2		.000		
Likelihood Ratio	50.935	2		.000		
No of Valid Cases		133				

Genetic counseling

The majority of parents (117, 88%) reportedly received genetic counseling after discovering their child had SCD while 16 (12%) did not (Table 7).

**Table 7:** Genetic counseling (a) Two cells (33.3%) have expected count less than 5. The minimum expected count is 3.73.

			Initial Genotype (Respondent + Spouse)			
			Correct Freq. (%)	Misidentified Freq. (%)	Unknown Freq. (%)	Total
Genetic Counseling	No		3 (9.7%)	6 (18.2%)	7 (10.1%)	16 (12.0%)
	Yes		28 (90.3%)	27 (81.8%)	62 (89.9%)	117 (88.0%)
Total			31 (23.3%)	33 (24.8%)	69 (51.9%)	133 (100.0%)
Chi-Square Tests						
		Value	df	Asymp. Sig. (2-sided)		
Pearson Chi-Square		1.574(a)	2	.455		
Likelihood Ratio		1.457	2	.483		
No of Valid Cases			133			

## Discussion

The findings show that the prevalence of a surprise diagnosis of SCD in children of unsuspecting and ill-prepared parents is high (52%). Parents and caregivers of children with SCD are faced with several challenges, which can be costly for the family and can result in a subpar health-related quality of life due to the parents’ ill-preparedness to finance a child’s healthcare [17].

Early diagnosis of SCD provides the opportunity to deliver early treatment and appropriate and timely vaccination and management [18]. A randomized controlled trial comparing early penicillin prophylaxis to the placebo was terminated eight months early due to the overwhelming evidence in support of the ability of penicillin to prevent pneumococcal sepsis in babies with SCD, with an 84% reduction in the rate of infection and the absence of fatalities in the penicillin prophylaxis study group [16]. The overall health of infants with SCD depends on the psychosocial preparedness of the parents [19].

Missed identification poses a significant impact on the type of care received. Several interventions introduced in early childhood, including penicillin prophylaxis, transcranial Doppler scans, and pneumococcal vaccination, have decreased the mortality rate [20]. The improved quality of life with the early introduction of analgesics, high fluid intake, folic acid supplementation, antibiotics, rest, and good nutrition is necessary to provide the best prospect for the child with SCD [21].

SCD has a considerable influence on the socioeconomic status of a family. Twenty-two percent of respondents reported having marital discord due to the stress of having a child with SCD; marital discord can further negatively influence the quality of care of their child with SCD due to potentially creating stressors at home for the child with SCD. Parents also reported a high incidence of their other children showing resentment towards their child(ren) with SCD (19%), again potentially increasing the stressors the child with SCD has to deal with it. Nevertheless, many parents also reported that having a child with SCD has brought the family closer together (47%), thus demonstrating that families can rally together to serve as a support system for one another. Respondents also frequently reported the frequent absences their child with SCD had from school. However, parents did not let it affect the school attendance of their other children (78.2%).

As mentioned earlier, SCD can have a significant impact on the finances of a household. Most of the parents reported a monthly household income of 10-30,000 naira. Though a majority of the respondents stated that they spend less than 2,000 naira/month for the general care of one child with SCD, financial hardship can easily arise when the child falls ill or needs to be hospitalized. According to respondents, most hospitalizations of a child with SCD cost more than 20,000 naira (60%), thus a large majority of the respondents of this study spend a range, from 60% of their monthly income to all of their income, with the need to also take out loans (43%) per hospitalization. Financial problems are compounded because many parents also have unstable employment. Though a majority of respondents reported that there was no influence on their employment due to having a child with SCD, 14.5% of respondents stated that they were voluntarily unemployed to care for a child with SCD, further reducing the income the household earns/month.

The study demonstrated that knowledge of SCD correlates with educational level and financial status. More-educated parents knew about SCD before having an affected child. The highest risk of being misidentified as a non-carrier of a hemoglobinopathy trait lies with those in a higher income bracket (> 60,000 Naira) and those with higher levels of formal education. Respondents in these classifications were less likely to have initially unknown genotypes and had an increased likelihood of using a private lab facility. Utilization of private lab facilities proves to be a precarious option due to the lack of oversight and quality control, which may contribute to the significantly higher rate of misidentification of genotypes (88%) as compared to those that occur in public facilities (12%). Inversely, it can be inferred that the lack of education and funds inadvertently protected from incorrect identification. However, the same group is shown to be at an increased risk due to ignorance and lack of preparation.

The majority of respondents who knew about SCD before the birth of their child with the disorder reported learning about the health condition through the hospital (50%), followed by school (17%). Surprisingly, media (TV, newspaper, radio, etc.) was the second least cited source of knowledge about the disorder (7%). Most respondents stated that school (34%) was the reason for their initial (before having a child with SCD) genotype testing, followed by awareness of the disorder (20%) and marital counseling (19%). This demonstrates the significant public health role that hospitals and school systems play in educating and mobilizing the public about the disorder. It is also important to note that many respondents quoted “antenatal care” (14.7%) as a reason for getting initial SCD genotyping done. This finding is significant, potentially correlating to the reason why more mothers than fathers are genotyped before having an affected child, also capturing another prospective technique to educate the population further.

Limitations

One of the limitations of the study was the inability or lack of access to interview both parents of each child, which resulted in questionable data regarding the income reported by the mother. Of the parents interviewed, mothers were the majority in the study; as a result, there is a biased motherly view of the data collected. Also, the educational status of the spouse was unable to be assessed, which inhibited the ability to categorize the social class of each participant. Another limitation of the study was the “Nigerian parlance,” encountered while administering the questionnaire. Due to cultural reasons, many parents would not want to speak negatively, as they do not want their statements to come to fruition as a result. Instead of answering questions truthfully, or candidly, parents would give answers such as “God will provide,” “It is well,” “The child is strong,” and “God forbid.” This occurrence slightly disturbed the data collection during the study.

Recommendations

The government should initiate an SCD awareness campaign, utilizing more media sources to reach the population, creating awareness and providing knowledge of SCD. A push to have schools and antenatal care providers strengthen SCD awareness and educative programs would also be a great benefit to society. Lastly and most importantly, the government should help standardize and improve the quality control of laboratory practices, especially in the private sector, by implementing policies and establishing oversight over the industry. This project would serve as a great pilot for a multicenter study to increase the power and reduce variance.

## Conclusions

SCD presents significant challenges to both the families of affected children and the country’s healthcare system. There is a high rate of missed information and misinformation about the risk of having children with SCD, leaving parents ill- or unprepared. Currently, private hospitals are less accurate at identifying hemoglobin genes relevant to SCD. Additionally, antenatal care and schools are great platforms to promote the testing and awareness of the disease. This study is the first to address the issue of missed identification and incorrect identification of sickle cell trait carriers in Nigeria. Our results demonstrate that there is room for cost-effective, public-health-oriented contributions to increase the awareness and implementation of screening for SCD. Furthermore, a greater push in ensuring that citizens receive accurate and affordable SCD genotyping results by establishing quality control standards. These measures can help combat the prevalence of the disease and empower potential future families to make informed decisions.

## Figures and Tables

**Figure 1:**
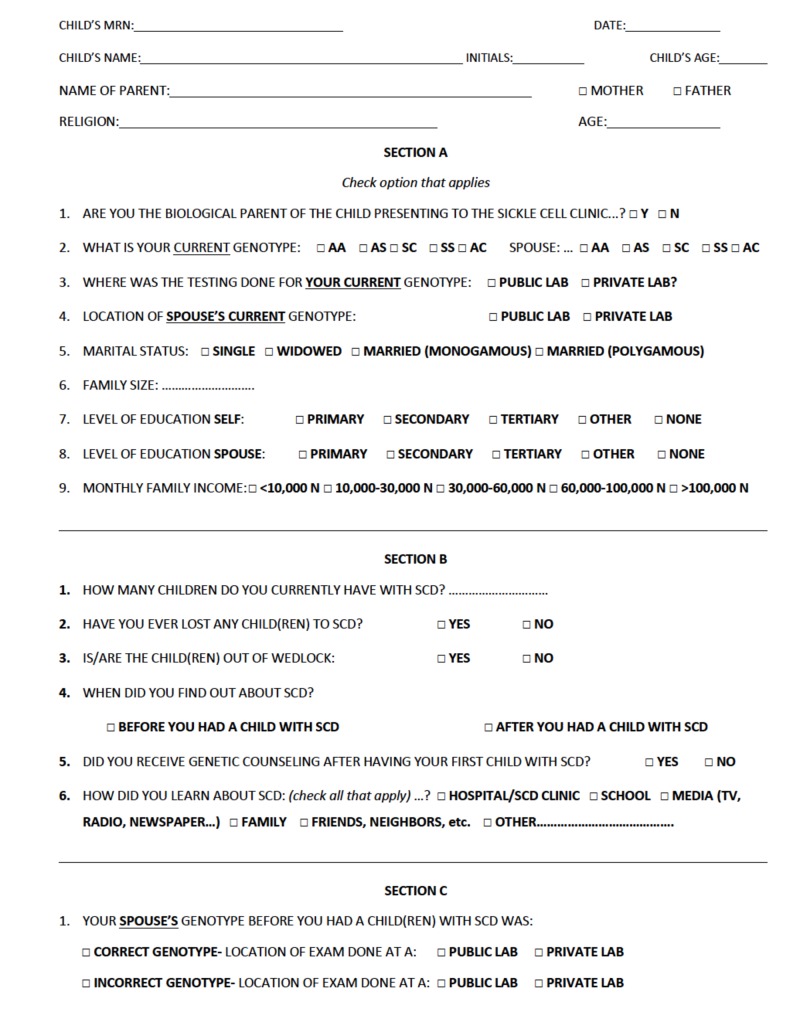
SCD questionnaire: page 1 SCD: sickle cell disease

**Figure 2:**
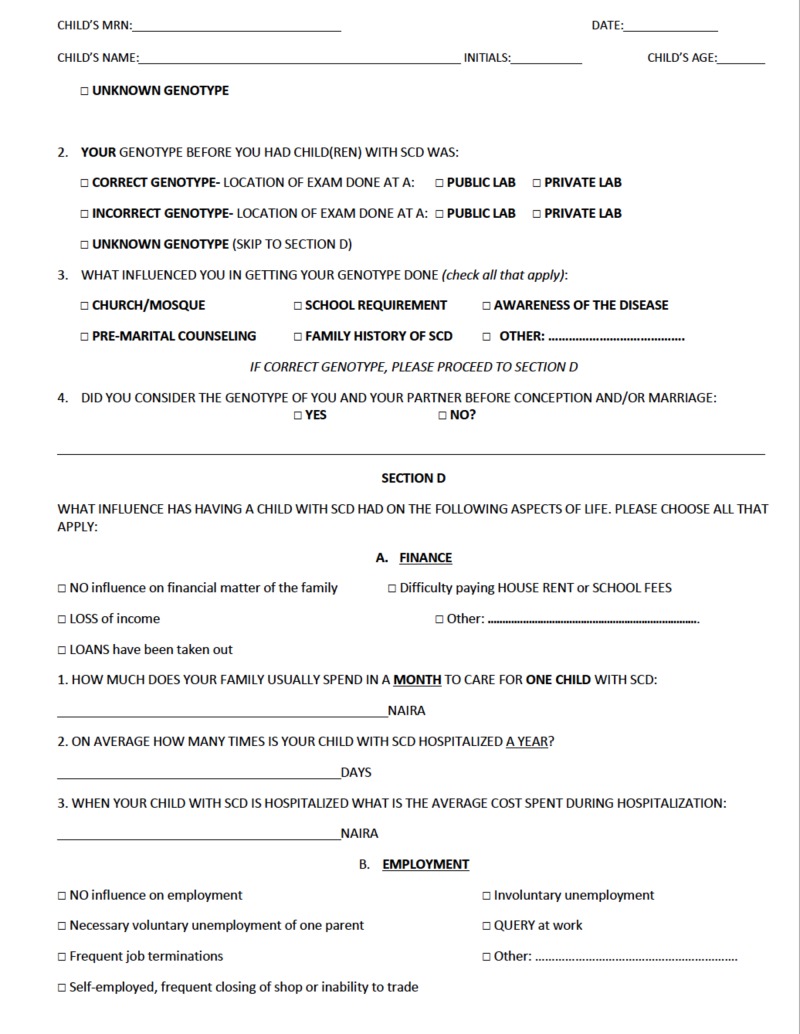
SCD questionnaire: page 2 SCD: sickle cell disease

**Figure 3:**
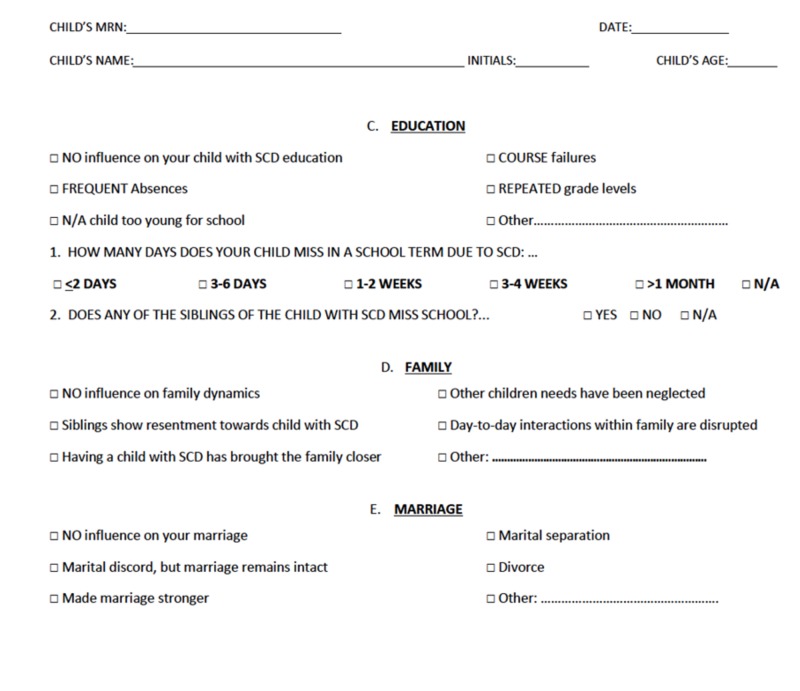
SCD questionnaire: page 3 SCD: sickle cell disease

## Appendices

Figures 1-3 show the semi-structured questionnaire created and used by the authors for the study:
